# Purification and Characterization of Nit*_*phym*_*, a Robust Thermostable Nitrilase From *Paraburkholderia phymatum*

**DOI:** 10.3389/fbioe.2021.686362

**Published:** 2021-07-01

**Authors:** Thomas Bessonnet, Aline Mariage, Jean-Louis Petit, Virginie Pellouin, Adrien Debard, Anne Zaparucha, Carine Vergne-Vaxelaire, Véronique de Berardinis

**Affiliations:** Génomique Métabolique, Genoscope, Institut François Jacob, CEA, CNRS, Univ Evry, Université Paris-Saclay, Evry, France

**Keywords:** heat treatment, substrate promiscuity, mesophilic organism, thermostability, nitrilase

## Abstract

Despite the success of some nitrilases in industrial applications, there is a constant demand to broaden the catalog of these hydrolases, especially robust ones with high operational stability. By using the criteria of thermoresistance to screen a collection of candidate enzymes heterologously expressed in *Escherichia coli*, the enzyme Nit*_*phym*_* from the mesophilic organism *Paraburkholderia phymatum* was selected and further characterized. Its quick and efficient purification by heat treatment is of major interest for large-scale applications. The purified nitrilase displayed a high thermostability with 90% of remaining activity after 2 days at 30°C and a half-life of 18 h at 60°C, together with a broad pH range of 5.5–8.5. Its high resistance to various miscible cosolvents and tolerance to high substrate loadings enabled the quantitative conversion of 65.5 g⋅L^–1^ of 3-phenylpropionitrile into 3-phenylpropionic acid at 50°C in 8 h at low enzyme loadings of 0.5 g⋅L^–1^, with an isolated yield of 90%. This study highlights that thermophilic organisms are not the only source of industrially relevant thermostable enzymes and extends the scope of efficient nitrilases for the hydrolysis of a wide range of nitriles, especially *trans*-cinnamonitrile, terephthalonitrile, cyanopyridines, and 3-phenylpropionitrile.

## Introduction

Nitriles are organic compounds of natural or synthetic origin that bear a cyano group known to be toxic and mutagenic to living organisms. Despite this feature, the versatility offered by this chemical function makes them key intermediates in the production of fine chemicals and pharmaceuticals. In addition to be precursors of various chemical functions such as amides and amines, their hydrolysis leads to the formation of carboxylic acids, important building blocks for organic synthesis. When carried out by conventional organic synthesis, this hydrolytic step requires strongly acidic or basic reaction conditions and high temperature, thus usually undergoing formation of unwanted byproducts and racemization in case of chiral compounds (Larock, 1989). Given their presence in the environment, some plants and microorganisms, such as filamentous fungi (Martínková, 2019) and more commonly bacteria (Nigam et al., 2017), have developed catabolic pathways for nitrile metabolization through the action of enzymes, such as nitrilases (EC 3.5.5.1) and nitrile hydratases (4.2.1.84). Nitrilases hydrolyze nitrile functions into their respective carboxylic acid functions in mild conditions, with high yield and selectivity. They are “easy” enzymes that do not require any cofactor or metal ions; thus, biocatalytical hydrolysis carried out by nitrilases tends to be preferred over chemical methods due to their efficiency and relative ecological friendliness (Singh et al., 2006; Gong et al., 2012; Nigam et al., 2017; Bhalla et al., 2018). This last decade, they have been successfully developed to facilitate the production of chemicals (Shen et al., 2021), such as acrylic acid (Nagasawa et al., 1990) and (*R*)-(-) mandelic acid (Fan et al., 2017b). Moreover, nitrilases have also found application in the surface modification of acrylic fibers in the textile industry (Matama et al., 2007) and in industrial bioremediation processes, such as degradation of the highly toxic 2,6-dichloro-benzonitrile and bromoxynil (3,5-dibromo-4 hydroxybenzonitrile) present in wastewater (Mueller et al., 2006; Nigam et al., 2017; Park et al., 2017).

Despite these successful examples, there is a constant demand for discovery of new robust nitrilases, the industrial application of reported nitrilases being limited by their global insufficient stability, their low tolerance to high substrate concentration, and/or their low specific activity (Bhalla et al., 2018). The operational stability of an enzyme is a key element for its use in biocatalysis on a commercial scale (Hauer, 2020), especially thermostability, as processes are often run at higher temperature than room temperature to increase substrate and product solubility and minimize the risk of microbial contamination (Vieille and Zeikus, 2001). Furthermore, thermostable biocatalysts are often tolerant to high pressure and solvents, experimental conditions often used in industrial processes. *Inter alia*, nitrilases satisfying these criteria are searched among the (hyper)thermophilic biodiversity (Atalah et al., 2019), through functional screenings (Soares Bragança et al., 2017) or (meta)genome mining approaches (Zhu et al., 2007; Seffernick et al., 2009; Vergne-Vaxelaire et al., 2013; Bordier et al., 2014; Vesela et al., 2016; Sharma et al., 2017; Egelkamp et al., 2020). Despite all the characterized nitrilases, no common feature of protein sequences was found that could explain their stability, only highly specific motifs were proposed as being important, thus making protein engineering complicated. Nevertheless, some nice examples have been reported to overcome stability limitations of nitrilases (Xu et al., 2018; Zhang et al., 2020) in addition to other engineering work targeting enantioselectivity/specificity or substrate inhibition at high loadings (Stolz et al., 2019).

Facing the industrial demand, new stable and efficient nitrilases tolerant to highly substrate concentration have still to be found to enrich this enzyme toolbox. Here we report the identification of a robust and thermostable nitrilase identified by the activity screening of a collection of 164 candidate nitrilases from various organisms before and after a heat treatment. This thermostable nitrilase from a mesophilic organism was characterized and appeared to be robust toward cosolvent and high substrate loadings with a large scope of substrates, demonstrating that thermophilic organisms are not the only source of industrially relevant thermostable enzymes.

## Materials and Methods

### Chemicals, Strains, and Materials

All reagents were purchased from commercial sources and used without additional purification. Terephthalonitrile, 2-cyanopyridine, 3-cyanopyridine, 4-cyanopyridine, 2,4-pyridine dicarbonitrile, 2,6-pyridine dicarbonitrile, benzonitrile, fumaronitrile, 4-hydroxybenzonitrile, 4-cyanobenzoic acid, crotonitrile, adiponitrile, valeronitrile, glutaronitrile, butyronitrile, propionitrile, isobutyronitrile, acetonitrile, 2-thiopheneacetonitrile, indol-3-acetonitrile, phenylacetonitrile, 3-phenylpropionitrile, 3-phenylpropionic acid, 3-phenylpropionamide, α-ketoglutarate, β-Nicotinamide adenine dinucleotide reduced (NADH), glutamate dehydrogenase (GDH), adenosine-diphosphate (ADP) were purchased from Sigma-Aldrich (Millipore Sigma, St. Louis, MO, United States). Buffers [sodium citrate, potassium phosphate, glycylglycine (Gly-Gly), 4-(2-hydroxyethyl)-1-piperazineethanesulfonic acid (HEPES), tris(hydroxymethyl)-aminomethane hydrochloride salt (Tris-HCl), glycine, sodium acetate, and 2-(*N*-morpholino)ethanesulfonic acid (MES)] were produced from substances purchased from Sigma–Aldrich (Millipore Sigma, St. Louis, MO, United States) and adjusted to the desired pH with sodium hydroxide (NaOH) for Gly-Gly, glycine, MES, and with hydrochloric acid (HCl) for Tris-HCl and HEPES. HPLC-UV analyses were performed on a Waters model 2795 liquid chromatograph provided with a Waters model 996 Photodiode Array Detector with a Kinetex C18 column (150 × 4.6 mm; 5 μm) (Phenomenex, CA, United States). UHPLC-UV analyses were performed on a UHPLC U3000 RS 1034 bar system (Thermo Fisher Scientific, Waltham, MA, United States) equipped with a UV detector, with a Kinetex EVO C18 column (100 mm × 2.1 mm; 1.7 μm) (Phenomenex, CA, United States). The spectrophotometric assays were recorded on a Safas UVMC2 (Safas, Monaco) thermostated when specified with a refrigerated/heating circulator Corio CD-200F (Jubalo^®^, Seelbach, Germany) using microcells high-precision cell quartz with 10- or 6-mm light path (Hellma Analytics, Müllheim, Germany). *Paraburkholderia phymatum* was from DSMZ collection (Leibniz Institute, Germany). NMR spectra were recorded on a Bruker (Bruker, Billerica, MA, United States) 600 MHz spectrometer (Evry University, France) for ^1^H and ^13^C experiments. Chemical shifts (expressed in ppm) of ^1^H and ^13^C spectra were referenced to the solvent peak δ(H) = 7.20 and δ(C) = 76.0 for CDCl_3_, respectively.

### Expression and Purification by Heat Treatment of the Nitrilase Collection

The nitrilase collection was produced as described in Vergne-Vaxelaire et al. (2013). Protein overexpression was carried out with *Escherichia coli* BL21-CodonPlus (DE3)-RIPL competent cells (Agilent Technologies, Santa Clara, CA, United States) to improve protein expression by overcoming codon bias. The 96-microwell plates containing cell crude extracts were heated at 70°C for 40 min in a thermocycler (GeneAmp PCR System 9700, Applied Biosystems, Foster City, CA, United States). After centrifugation, the supernatants were deposited on a sodium dodecyl sulfate–polyacrylamide gel electrophoresis (SDS-PAGE) gel using the E-PAGE High-Throughput system (Invitrogen).

### Screening Assay of the Nitrilase Collection

The 96-microwell plates containing cell crude extracts of the nitrilase collection were tested for their hydrolytic activity toward fumaronitrile, glutaronitrile, and 3-phenylpropionitrile. All the reactions were conducted in 96-microwell plates in 100 μL reaction volume containing 10 mM nitrile, 100 mM potassium phosphate buffer pH 7.5, 1 mM DTT and 10 μL of crude cell extract (or 20 μL of supernatant resulting from the heat treatment). The reaction was carried out at 30°C for 4 h or at 50°C for 1 h; 100 μL of a mixture containing 100 mM potassium phosphate buffer pH 7.5, 300 μM NADH, 100 μM ADP, 10 mM α-ketoglutaric acid, and 3.15 U⋅mL^–1^ of GDH were next added. The oxidation of NADH was monitored by spectrophotometry at 340 nm to evaluate the release of NH_3_, as previously reported (Vergne-Vaxelaire et al., 2013). An active enzyme corresponds to a microwell exhibiting a higher slope (0–120 s) over a background microwell without substrate.

### Large Scale Purification of Nit*_*phym*_*

Large-scale purification of Nit*_*phym*_* was conducted from a 400 mL culture by nickel affinity chromatography (His Trap FF 5 mL) in tandem with gel filtration (Hi Load 16/60 Superdex 200 pg) as described elsewhere (Perchat et al., 2017). The storage buffer was 50 mM Tris-HCl pH 8.0, 50 mM NaCl, 15% glycerol, and 1 mM DTT. Protein concentration was determined by the Bradford method with bovine serum albumin as the standard (Bradford, 1976). The sample was analyzed by SDS-PAGE using the Invitrogen NuPAGE system. The purified protein was stored at −80°C.

### Determination of Oligomerization State

Analytical gel filtration was performed on a Superdex 200 Increase 10/300 GL column by injection of 100 μL of the purified protein (3.9 mg⋅mL^–1^) with the following mobile phase: 50 mM Tris-HCl pH 8.0, 0.15 mM NaCl, 10% glycerol at a flow rate of 0.3 mL⋅min^–1^. The oligomerization state of Nit*_*phym*_* was determined according to its elution volume compared to a calibration curve obtained with proteins with known molecular weight.

### Purification of Nit_*phym*_ by Heat Treatment

Cell free extract from a 200 mL culture was lysed as previously described (Bastard et al., 2014). After centrifugation, the supernatant was heated for 20 min at 70°C. Supernatant was then recovered and protein concentration was determined by the Bradford method with bovine serum albumin as standard (Bradford, 1976). The sample was analyzed by SDS-PAGE using the Invitrogen NuPAGE system. The purified protein was stored at -80°C.

### Specific Activity of Nit_*phym*_

The specific activities were determined by spectrophotometry using a modified protocol of the GDH/α-ketoglutaric acid assay described above. To a mixture heated to 40°C (100 μL, microcell 6-mm light path) containing 100 mM potassium phosphate buffer pH 8.1, 10 mM of nitrile, 1 mM DTT, and for enzymatic coupling assay 10 mM α-ketoglutarate, 0.25 mM NADH, 0.1 mM ADP, and 8 μg of GDH enzyme was added the appropriate amount of Nit*_*phym*_* purified by nickel affinity chromatography. Specific activity of Nit*_*phym*_* purified by heat treatment was assayed on 3-phenylpropionitrile to compare the activity of the two enzymes. The specific activities were determined from duplicate experiments by monitoring the consumption of the NADH at 340 nm. The background noise was estimated from blank reaction lacking substrate.

### HPLC-UV and UHPLC-UV Conditions

Condition A: The reaction mixtures were analyzed by HPLC-UV (eluent MeCN/H_2_O + 0.1% HCO_2_H with an isocratic mode 30/70 during 10 min); flow rate 1.5 mL⋅min^–1^; temperature 30°C; injection volume 10 μL; diode array detection λ = 210–400 nm. Conversions were deduced from the ratio of integrated substrate area and integrated product area as 3-phenylpropionitrile and 3-phenylpropionic acid displayed similar UV response over the whole range 210–400 nm.

Condition B: The reaction mixtures were analyzed by UHPLC-UV (eluent MeCN/H_2_O + 0.1% HCO_2_H with a linear gradient 20/80 during 2 min, then 20/80–70/30 in 5 min followed by re-equilibration time); flow rate 0.5 mL⋅min^–1^; temperature 25°C; injection volume 3 μL; UV detection at λ = 220 nm). The retention times of 3-phenylpropionitrile, 3-phenylpropionic acid, and 3-phenylpropionamide were 2.90, 2.32, and 1.27 min, respectively. The conversions were deduced from calibration curves obtained with commercial substrate and product as standards.

### Characterization of Nit_*phym*_

All reactions mixtures (100 μL) containing 100 mM potassium phosphate buffer pH 7.5 (or others buffers for the study of the effect of pH and buffer), 10 mM 3-phenylpropionitrile, 10% (vol/vol) methanol, 1 mM DTT were stirred at 600 revolutions/min (rpm). Each reaction was stopped at the indicated time by addition of 1% (vol/vol) TFA and analyzed after centrifugation for 5 min at 13,000 rpm and filtration (0.22 μm).

Thermostability was determined by monitoring the conversion of 3-phenylpropionitrile into 3-phenylpropionic acid by HPLC-UV according to condition A. Samples were incubated at 30°C for 20 min with enzyme preincubated at the specified temperature (30, 40, 50, 60, 70, and 80°C) for the specified duration (0–48 h). Reactions contained 0.16 mg⋅mL^–1^ Nit*_*phym*_* purified by heat treatment.

Conversion, thermoactivity, effect of pH, buffer, and tolerance to water-miscible cosolvent were determined by monitoring the conversion of 3-phenylpropionitrile into 3-phenylpropionic acid by UHPLC-UV according to condition B. Reactions contained 0.07 mg⋅mL^–1^ Nit*_*phym*_* purified by heat treatment.

1. Conversion: samples were incubated at 30°C for the indicated duration (0–180 min)
2. Thermoactivity: samples were incubated at different temperatures (10, 20, 30, 40, 45, 50, 55, 60, 70, and 80°C) for 20 min.
3. Effect of buffer and pH: sodium acetate buffer (pH 4.0–5.5), sodium citrate buffer (pH 4.4–5.3), MES-NaOH buffer (pH 5.5–6.5), Tris-HCl buffer (pH 7.0–9.0), Gly-Gly-NaOH buffer (pH 7.5–8.5), potassium phosphate buffer (pH 6.3–8.3), HEPES-HCl buffer (pH 7.0–7.5), and glycine-NaOH buffer (pH 9.0–10.0) were tested. Reactions were incubated at 30°C for 20 min.
4. Effect of water-miscible cosolvent: various ratio [1, 5, 10, 15, 20, 30, and 40% (vol/vol)] of methanol (MeOH), acetonitrile (MeCN), dimethylsulfoxide (DMSO), isopropanol (iPrOH), and tetrahydrofuran (THF) were tested. Reactions were incubated at 50°C for 20 min.

### Effect of Substrate Loadings on Conversion of 3-Phenylpropionitrile

The tolerance of Nit*_*phym*_* to high substrate concentrations was studied by monitoring the conversion of 3-phenylpropionitrile into 3-phenylpropionic acid at 50°C for 48 h. Reaction mixtures (100 μL), containing 100 mM of potassium phosphate buffer pH 7.5, 1 mM DTT, 0.1 mg⋅mL^–1^ of Nit*_*phym*_*, and 3-phenylpropionitrile at a concentration range of 100–600 mM with 20% (vol/vol) of DMSO, were incubated at 50°C at 400 rpm. Samples were then diluted five times (vol/vol) in water and conversion of 3-phenylpropionitrile was determined by UHPLC-UV assay (condition B).

### Preparative Scale Reaction

In a 50 mL-Greiner tube equipped with a screw cap was poured 3-phenylpropionitrile (6 mL of a 2.5 M stock solution in DMSO, 15 mmol), distilled water (12.2 mL), potassium phosphate buffer pH 7.5 (3 mL of a 1 M stock solution) and Nit*_*phym*_* purified by heat treatment (8.8 mL of a 0.5 mg⋅mL^–1^ stock solution). The reaction was shaken at 50°C 500 rpm with UHPLC-UV monitoring (condition B). After 8 h, the reaction was quenched by 1% (vol/vol) TFA and centrifuged (5 min, 4,000 rpm, 4°C) to remove the proteins. The pellet was washed with distilled water (2 × 10 mL, centrifugation 5 min, 4,000 rpm, 4°C) and the combined supernatants were extracted with Et_2_O (2 × 30 mL) after addition of concentrated HCl to aqueous phase until pH 1–2. The combined organic layers were dried (MgSO_4_), filtered, and concentrated under reduced pressure to give 2.02 g of the desired 3-phenylpropionic acid as a white solid, yield 90%.

## Results

### Identification of a Thermostable Nitrilase From a Nitrilase Collection

**FIGURE 1:**
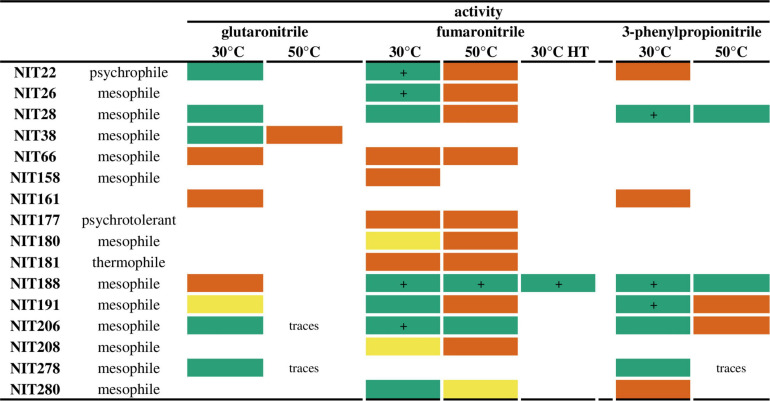
Activity of the nitrilase collection at 30, 50°C, and after heat treatment (HT). Reaction conditions: 10 mM nitrile, 100 mM potassium phosphate buffer pH 7.5, 1 mM DTT, 10 μL of crude cell extract, or 20 μL of supernatant resulting from the heat treatment, 30°C for 4 h or at 50°C for 1 h. These qualitative results correspond to the corrected slope obtained by the spectrophotometric coupled GDH activity assay: green box = | slope| > 100 abs/min (“+”: very high activity as all the NADH is consumed in the first few seconds after addition of the crude cell lysate); yellow box = 40 ≤ | slope| < 100 abs/min; orange box = 5 ≤ |slope| < 40 abs/min. Only the enzymes displaying activity on at least one of these substrates are listed in this figure. For more details, see “Materials and Methods.”

We previously reported a collection of 164 heterologous overexpressed nitrilases, selected from biodiversity by a sequence-driven approach (Vergne-Vaxelaire et al., 2013). Cell free extracts were produced and activities toward glutaronitrile, fumaronitrile, and 3-phenylpropionitrile were tested in 96-microwells at 30 and 50°C. These nitriles were reported to be the best substrates of this collection. Glutamate dehydrogenase/α-ketoglutarate was used as enzymatic couple spectrophotometric assay to monitor at 340 nm the oxidation of NADH that reflects indirectly NH_3_ liberated by the nitrile hydrolysis (Vergne-Vaxelaire et al., 2013). The concentrations of proteins are of the same order of magnitude in each microwell, enabling qualitative analysis of the activity results reported in Figure 1. All the crude cell lysates displaying high activities at 30°C have shown much lower activities at 50°C, excepting NIT188 and NIT206, both coming from mesophilic organisms. These two overexpressed enzymes were also ones of the most active enzymes at 30°C toward the tested substrates, particularly fumaronitrile and 3-phenylpropionitrile, together with NIT22, NIT26, NIT28, and NIT191. The same cell free extracts were then submitted to a heat treatment (70°C for 40 min). After centrifugation, the supernatants were tested for their nitrilase activity at 30°C, only toward fumaronitrile. Indeed, fumaronitrile was substrate of all the enzymes still showing an activity at 50°C and so potentially more thermostable. The only exception NIT38 displayed a narrow substrate spectrum and only a moderate activity toward its sole substrate glutaronitrile. Only NIT188 displayed an activity both before and after heat treatment. Interestingly, the activity at 30°C after heat treatment was of the same order of magnitude as before, and NIT188 was also one of the best enzyme in terms of activity and substrate range. This nitrilase from *P. phymatum DSM* 17167 (Uniprot ID: B2JQY2) is named Nit*_*phym*_*. The sole enzyme from a thermophilic organism, active toward the tested substrate in these conditions (NIT181), did not show better activity at 50°C compared to 30°C and did not display any activity after heat treatment.

### Characterization of Nit_*phym*_

**FIGURE 2:**
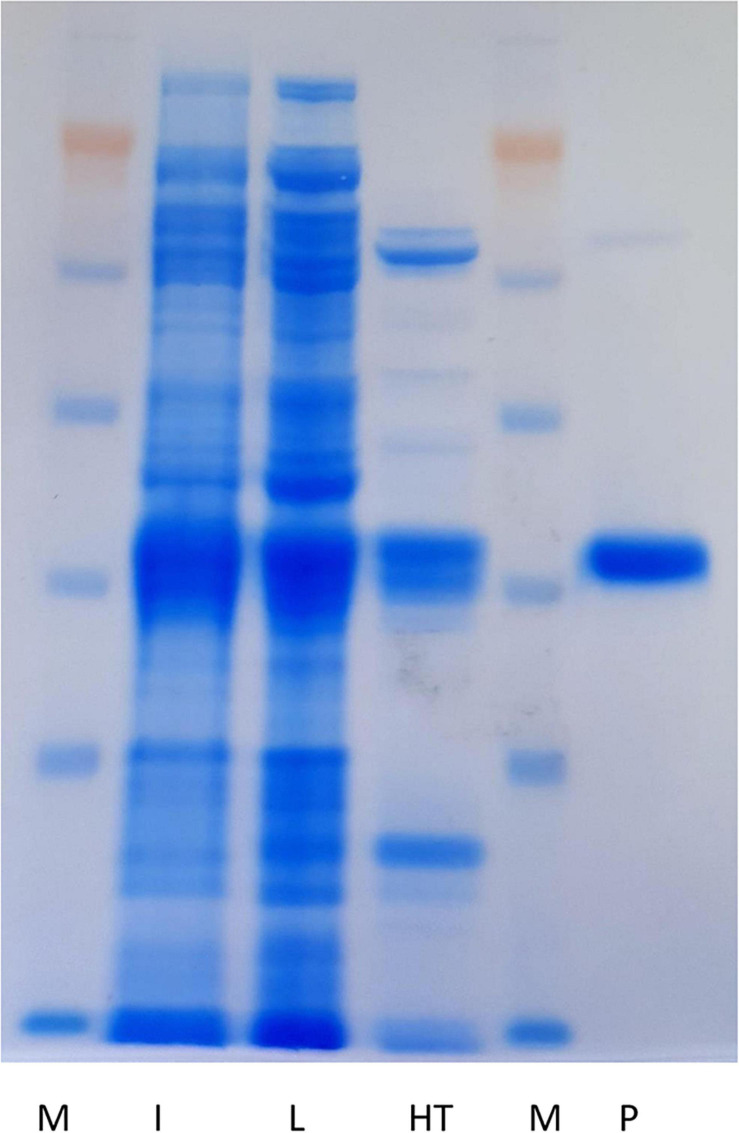
Electrophoretic analysis (SDS-PAGE using NuPAGE system of Invitrogen, 10%) of the purified Nit*_*phym*_*. M: protein molecular marker; I: induction (10 μL); L: soluble cell crude extract (approximately 15 μg); HT: supernatant after heat treatment (70°C for 20 min) (approximately 5 μg); P: purified nitrilase by nickel affinity chromatography in tandem with gel filtration (approximately 5 μg).

To confirm the thermotolerance previously observed in 96-microwells, cell crude extract, obtained from 200 mL of culture of recombinant *E. coli* overexpressing Nit*_*phym*_*, was submitted to a heat treatment (70°C for 20 min). In addition, 400 mL of culture were submitted to purification by nickel affinity chromatography in tandem with gel filtration for further activity comparison. After centrifugation, the heat shocked supernatant was deposited on a SDS-PAGE gel exhibiting a prominent band with an apparent molecular mass of 37 kDa (Figure 2), as for the one purified by gel filtration. This result is in agreement with the predicted molecular mass of the subunit. An analytical gel filtration experimentation has revealed an active oligomer having more than 18 subunits. Activities of both purified enzymes were calculated by the enzymatic couple spectrophotometric assay run with one of its validated substrate, i.e., 3-phenylpropionitrile. The specific activity of Nit*_*phym*_* purified by heat treatment was approximately twice lower as to the one measured with the batch purified by nickel affinity chromatography in tandem with gel filtration (0.98 ± 0.11 U⋅mg^–1^ and 1.90 ± 0.14 U⋅mg^–1^, respectively). However, the uncertainty of the amount of nitrilase due to the difference in purity of the two batches has to be kept in mind. It leads to an underestimation of the activity of the enzyme obtained by heat treatment, which proved to be purified with less efficiency (Figure 2). Longer heat treatment times did not improve purification and significantly decrease the activity of the enzyme (data not shown).

**FIGURE 3:**
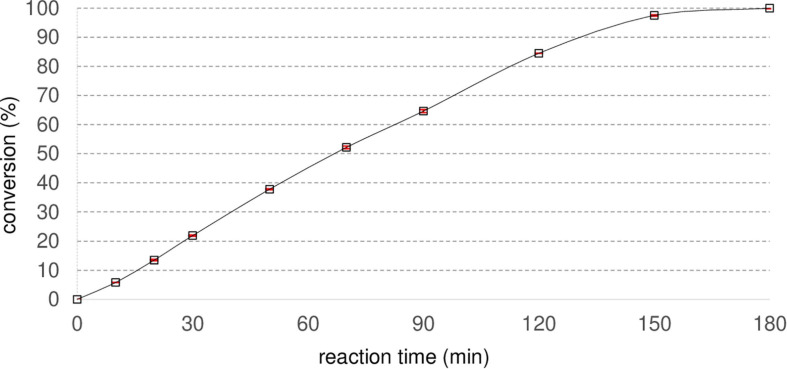
Conversion of 3-phenylpropionitrile into 3-phenylpropionic acid by Nit*_*phym*_* purified by heat treatment. Reaction conditions: 10 mM 3-phenylpropionitrile, 100 mM potassium phosphate buffer pH 7.5, 1 mM DTT, 0.07 mg⋅mL^– 1^ Nit*_*phym*_*, 600 rpm, 30°C. Reactions were analyzed by UHPLC-UV with condition B. Red error bars represent the standard deviation of two independent experiments.

To evaluate the biocatalytic capability of Nit*_*phym*_* purified by heat treatment, hydrolysis of 10 mM of 3-phenylpropionitrile at 30°C was studied by monitoring the UV active product 3-phenylpropionic acid formation by UHPLC-UV analysis over 180 min (Figure 3). In these reaction conditions, Nit*_*phym*_* converted 50% of 10 mM 3-phenylpropionitrile in 70 min and complete conversion was reached in 180 min. To characterize the nitrilase, the main parameters influencing the rate of the biocatalytic reaction were studied: thermostability, optimal temperature, pH, buffer nature, and tolerance to water-miscible cosolvent. All these tests were performed by HPLC-UV or UHPLC-UV after preliminary studies ensuring that they were carried out within the kinetic operating times of the enzyme. Substrate spectrum and tolerance to high substrate loadings were also determined to evaluate its potential as biocatalyst for hydrolysis of various nitriles.

### Thermostability and Optimal Temperature of Nit_*phym*_

**FIGURE 4:**
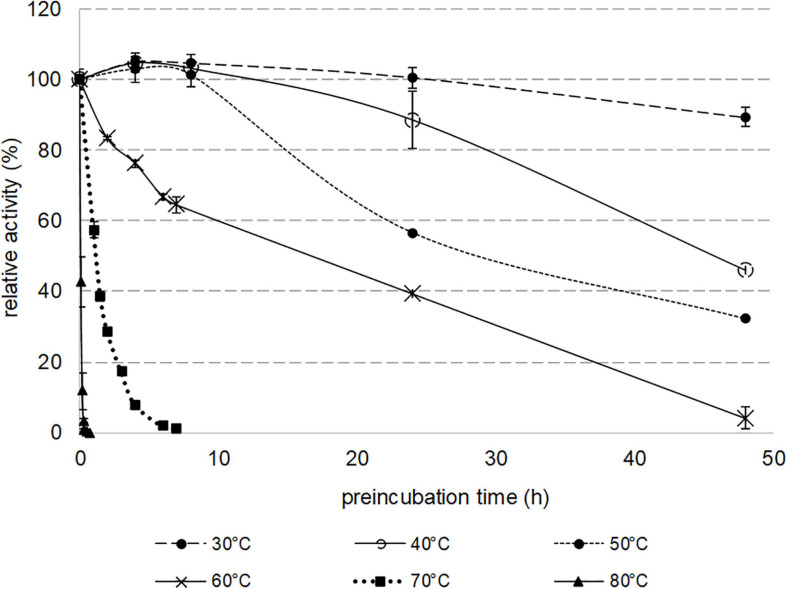
Thermostability of purified nitrilase Nit*_*phym*_*. Reaction conditions: 10 mM 3-phenylpropionitrile, 100 mM potassium phosphate buffer pH 7.5, 1 mM DTT, 0.16 mg⋅mL^– 1^ Nit*_*phym*_* preincubated at 30, 40, 50, 60, 70, and 80°C for various times (0–48 h), 600 rpm, 30°C, 20 min. The residual activities were calculated using HPLC-UV assay (condition A). The residual maximum activity obtained without preincubation (0.8 U⋅mg^– 1^) was set up at 100%. Error bars represent the standard deviation of two independent experiments.

Thermostability was assessed by analyzing the residual activity of Nit*_*phym*_* purified by heat treatment, toward 3-phenylpropionitrile after incubation of the enzyme at various temperatures from 30 to 80°C (Figure 4). The specific activity of the recombinant nitrilase was not affected after 7 h of incubation at 30–50°C. Incubations at temperatures greater than 70°C led to rapid loss of its activity, with a total lost after 20 min at 80°C. Notably, Nit*_*phym*_* was very stable at 30°C since, after 2 days of incubation, 90% of the initial activity remained. It showed high thermostability with half-lives of 18, 28, and 45 h at 60, 50, and 40°C, respectively.

**FIGURE 5:**
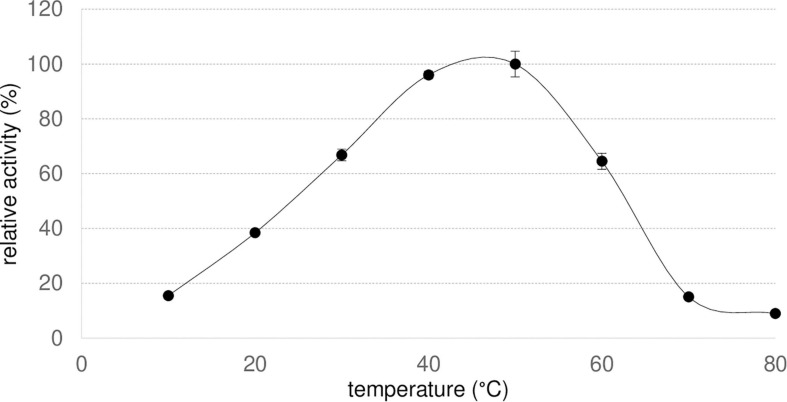
Optimal temperature of Nit*_*phym*_*. Reaction conditions: 10 mM 3-phenylpropionitrile, 100 mM potassium phosphate buffer pH 7.5, 1 mM DTT, 0.07 mg⋅mL^– 1^ Nit*_*phym*_*, 600 rpm, 10–80°C, 20 min. The activities were calculated using UHPLC-UV assay (condition B). The maximum activity obtained at 50°C (2.0 U⋅mg^– 1^) was set up at 100%. Error bars represent the standard deviation of two independent experiments.

To evaluate the optimal temperature of Nit*_*phym*_*, hydrolysis of 3-phenylpropionitrile was monitored at different temperatures from 10 to 80°C for 30 min using UHPLC-UV assay conditions (Figure 5). The reaction rate increased with temperature until an optimum at 40–50°C, and declined sharply at higher temperatures even if Nit*_*phym*_* kept around 65% of activity at 60°C.

### Substrate Specificity of Nit_*phym*_

We previously described qualitative activities of purified Nit*_*phym*_* toward various nitriles (Vergne-Vaxelaire et al., 2013). For a better characterization of this robust enzyme, its substrate spectrum was studied by spectrophotometry in microcuvettes using optimized GDH assay at 40°C and Nit*_*phym*_*, purified by affinity (Table 1). The 24 commercially available nitriles tested were chosen to cover a wide structural diversity, ranging from benzylic/unsaturated nitriles (entries 1–12), 3-phenylpropionitrile (entry 13), aliphatic nitriles (entries 14–20), and arylacetonitriles (entries 21–24). The α/β-hydroxyl- or amino-nitriles were not tested due to previously reported inactivity toward these substrates (Vergne-Vaxelaire et al., 2013). The data indicate that Nit*_*phym*_* can hydrolyze a broad range of nitriles, either benzylic/unsaturated ones like the preferred *trans*-cinnamonitrile (entry 1) and mono/di-cyanopyridine (entries 3–7), or aromatic substituted nitriles with a moderate activity toward 3-phenylpropionitrile (16% compared to 100% for *trans*-cinnamonitrile). Some activity was detected with 2-thiopheneacetonitrile but no other tested arylacetonitriles showed noticeable activity. Notably, this enzyme catalyzed the hydrolysis of aliphatic dinitriles like adiponitrile (entry 14) and in less extent glutaronitrile (3% compared to 100% for *trans*-cinnamonitrile). Fumaronitrile is a quite poor substrate among the substrate range. The high activity toward this substrate, observed during the heat treatment activity assay run in 96-microwell plates, illustrated the very strong activity of this enzyme on a wide range of substrates. Despite the moderate activity toward 3-phenylpropionitrile compared to other preferred nitriles, the later was used to further study this enzyme for its easy monitoring by UHPLC-UV, its good solubility, and its mention in many publications relating to nitrilases.

**TABLE 1:** Substrate specificity of Nit*_*phym*_.*

Entry	Substrate	Relative activity (%)
benzylic/unsaturated nitriles		
1	*trans*-Cinnamonitrile	100
2	Terephthalonitrile	53.6 ± 2.8
3	2-Cyanopyridine	32.1 ± 0.7
4	3-Cyanopyridine	30.0 ± 1.9
5	4-Cyanopyridine	24.1 ± 0.4
6	2,4-Pyridine dicarbonitrile^*a*^	19.8 ± 2.5
7	2,6-Pyridine dicarbonitrile^*a*^	11.8 ± 1.1
8	Benzonitrile	0.9 ± 0.1
9	Fumaronitrile^*a*^	2.3 ± 0.4
10	4-Hydroxybenzonitrile	<1
11	4-Cyanobenzoic acid	nd
12	Crotonitrile	1.5 ± 0.2
13	3-Phenylpropionitrile	16.2 ± 1.2
**aliphatic nitriles**		
14	Adiponitrile^*a*^	8.4 ± 0.3
15	Valeronitrile	5.2 ± 0.2
16	Glutaronitrile^*a*^	2.4 ± 0.4
17	Butyronitrile	1.6 ± 0.1
18	Propionitrile	<1
19	Isobutyronitrile	nd
20	Acetonitrile	nd
**arylacetonitriles**		
21	2-Thiopheneacetonitrile	3.3 ± 0.2
22	Indole-3-acetonitrile	<1
23	Phenylacetonitrile	<1
24	2,4-Isobutylphenylpropionitrile	<1

*^a^Dinitriles; nd, not detected. The nitrilase activity was assayed with 10 mM substrate under the optimized GDH assay conditions. The maximum activity obtained with trans-cinnamonitrile (9.3 U⋅mg^–1^) was set up at 100%. Data are reported as the average ± standard deviation of two or three independent experiments.*

### Effect of Buffer and pH on Nit_*phym*_ Relative Activity

**FIGURE 6:**
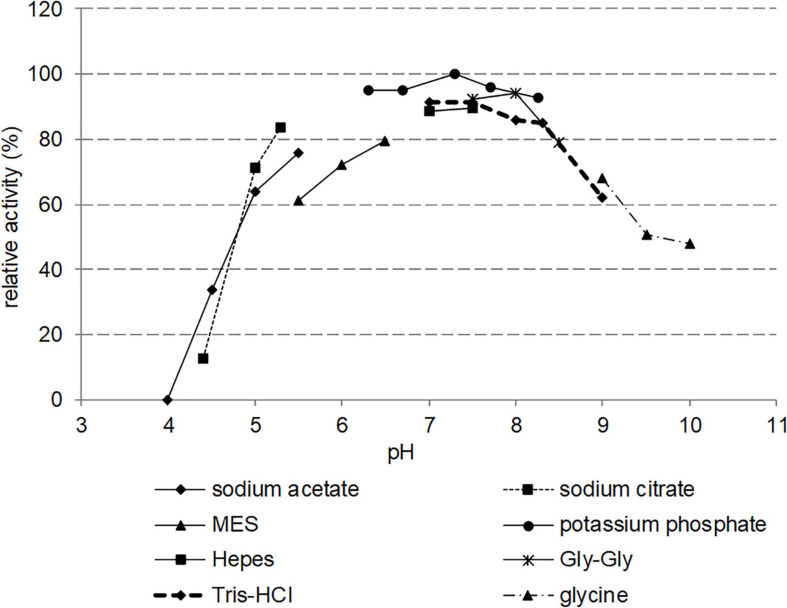
Effect of pH on the activity of Nit*_*phym*_*. Reaction conditions: 10 mM 3-phenylpropionitrile, 1 mM DTT, 0.07 mg⋅mL^– 1^ Nit*_*phym*_*, 600 rpm, 30°C, 20 min, in different buffers at pH ranging from 4.0 to 10.0. The activities were calculated using UHPLC-UV assay (condition B). The maximum activity obtained in potassium phosphate buffer at pH 7.3 (1.16 U⋅mg^– 1^) was set up at 100%.

The effect of pH was examined over a range of 4.0–10.0 (Figure 6). Various buffers were used to ensure buffering capacity: sodium citrate, potassium phosphate, Gly-Gly, HEPES, Tris-HCl, glycine, sodium acetate, and MES. Nit*_*phym*_* displayed a relatively broad pH profile with a maximum activity in potassium phosphate buffer at pH 6.0–8.0. Activities dropped at pH values below 5.5 and above 9.0. The nature of the buffer did not affect significantly the activity, only MES buffer induced approximately a 15% loss of activity compared to others tested buffers.

### Effect of Organic Water-Miscible Cosolvents on Nit_*phym*_ Activity

**FIGURE 7:**
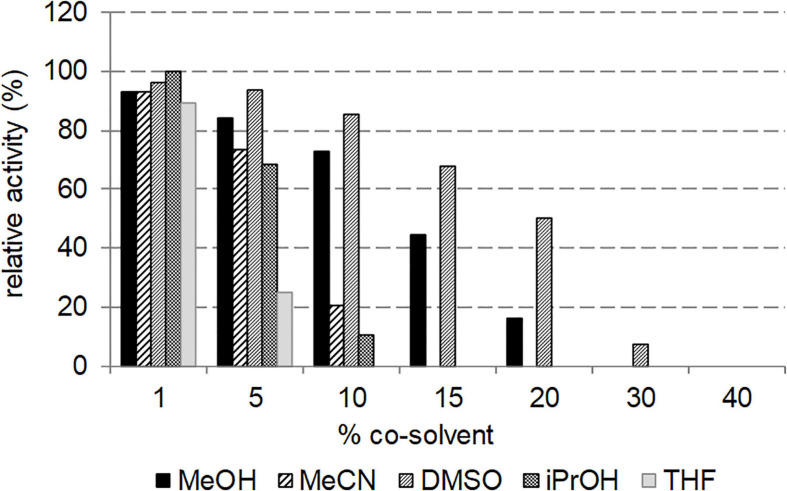
Effect of cosolvent on the activity of Nit*_*phym*_*. Reaction conditions: 10 mM 3-phenylpropionitrile, 100 mM potassium phosphate buffer pH 7.5, 1 mM DTT, 1–40% (vol/vol) of MeOH, MeCN, DMSO, iPrOH and THF, 0.07 mg⋅mL^– 1^ Nit*_*phym*_*, 600 rpm, 50°C, 20 min. The activities were calculated using UHPLC-UV assay (condition B). The maximum activity obtained with 1% of iPrOH (4.9 U⋅mg^– 1^) was set up at 100%.

The effect of various water-miscible cosolvents on the activity of Nit*_*Phym*_* was studied at 50°C at different volumetric percentage, using UHPLC-UV condition assay with 3-phenylpropionitrile as substrate. The studied solvents were dimethylsulfoxide (DMSO), methanol (MeOH), acetonitrile (MeCN), tetrahydrofuran (THF) and isopropanol (iPrOH) (Figure 7). All the tested organic solvents allowed similar Nit*_*phym*_* activity at 1% (vol/vol). MeOH and even more DMSO still enabled moderate to high activity until 15% (vol/vol). Notably for DMSO, more than 80 and 60% of relative activity could be observed in presence of 10 and 15% (vol/vol), respectively, and Nit*_*phym*_* still retained 50 and 7% of activity at 20 and 30% (vol/vol), respectively. The activity dramatically decreased at minimum 5% (vol/vol) THF (25% relative activity), 10% MeCN (20% relative activity), and iPrOH (10% relative activity), to become undetectable above 15% (vol/vol) for these three cosolvents.

### Effect of 3-Phenylpropionitrile Concentration on Conversion

**FIGURE 8:**
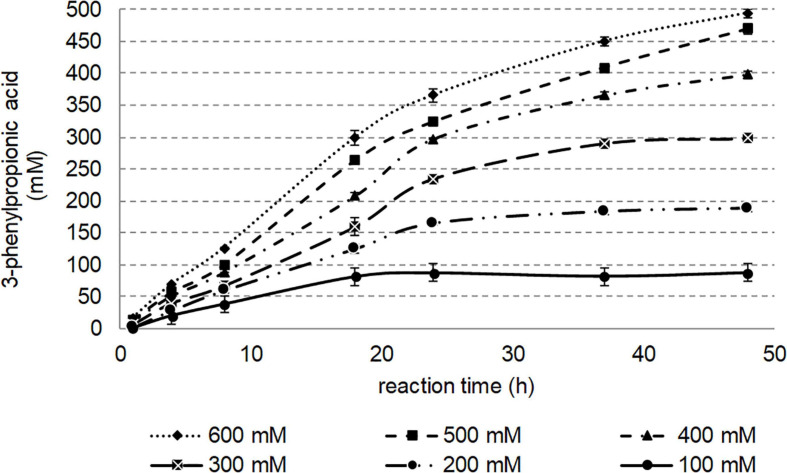
Effect of 3-phenylpropionitrile concentration on conversion. Reaction conditions: 100–600 mM 3-phenylpropionitrile, 100 mM potassium phosphate buffer pH 7.5, 1 mM DTT, 20% (vol/vol) DMSO, 0.1 mg⋅mL^– 1^ Nit*_*phym*_*, 400 rpm, 50°C, 48 h. The amount of 3-phenylpropanoic acid formed was determined by UHPLC-UV monitoring (condition B) and deduced from calibrations curves.

To determine the effect of substrate concentration, the reaction was performed at various substrate loadings (10–600 mM) over 48 h at 50°C, and the formation of 3-phenylpropionic acid was monitored by UHPLC-UV (Figure 8). Such substrate concentrations were reached thanks to 20% (vol/vol) of DMSO, this volume percentage being tolerated by Nit*_*phym*_*. To avoid any solubility issues which may be encountered with the samples taken from a reaction mixture at these high concentrations, distinctive batches were run and treated separately before UHPLC-UV analysis. Reasonable amount of enzyme (0.1 mg⋅mL^–1^) was used for this study to demonstrate the high potentiality of this enzyme for biocatalysis. Obviously, shorter conversion times can be obtained using higher amount of enzyme. As shown in Figure 8, after 18 h of incubation at 50°C, 100 mM of 3-phenylpropionitrile were nearly completely converted to the corresponding acid. Substrate concentrations as high as 500 mM can be nearly entirely converted in maximum 48 h in these conditions. No substrate inhibition can be observed in this study at concentration up to 600 mM, as for the same incubation time, higher amount of acid was produced when the nitrile concentration was increased.

### Preparative Scale Reaction With 3-Phenylpropionitrile

To evaluate the catalytic performance of Nit*_*phym*_*, a preparative scale reaction was performed with the enzyme purified by heat treatment (0.5 mg L^–1^) under the optimized conditions. 500 mM of 3-phenylpropionitrile was almost completely converted to 3-phenylpropionic acid at pH 7.5 and 50°C within 8 h at 30 mL final volume with 20% (vol/vol) DMSO (Supplementary Figure 1). The desired product was obtained by acidification, centrifugation, and extraction in Et_2_O as a white solid in 90% yield. The product was identical to the commercial standard as checked by ^1^H and ^13^C NMR (Supplementary Figures 2, 3). Purity of the isolated product was 99% as determined by UHPLC-UV analysis (Supplementary Figure 1).

## Discussion

The exploration of biocatalytic natural biodiversity is a very efficient strategy to provide enzymes with various features (Jeffries et al., 2016; Zaparucha et al., 2018). In addition to the approach used to select candidate enzymes among biodiversity, diverse conditions can also be used in the experimental screening to directly target the desired properties. In this study, starting from an already described enzyme collection, an alternative activity assay based on the resistance of the enzymes to temperature was performed to catch rapidly active and robust enzymes that can be easily purified by heat treatment. The protocol used was not suitable for nitrilases active at temperatures much higher than 50°C, and so to some nitrilases from extremophiles. The main objective remained to select enzymes that are robust but active at moderate temperatures to meet common industrial criteria. Operational stability is one of the key requirement for biocatalyst on a commercial scale.

We characterized the enzyme satisfying this criteria of resistance to heat treatment fixed in this study, namely the nitrilase from *P. phymatum* (Nit*_*phym*_*) previously identified from biodiversity (NIT188) (Vergne-Vaxelaire et al., 2013). We showed that even if this microorganism is a mesophilic one, the nitrilase is still in an active form after a heat treatment at 70°C, compared to other nitrilases of the collection active toward the tested substrates. An activity test toward the resulting supernatants seemed preferable to a study of the associated SDS-PAGE gels, bacterial nitrilases of this collection displaying moderate overexpression in crude cell lysates thus making gels difficult to analyze. Despite a decrease in activity of about a factor of 2 on the substrate 3-phenylpropionitrile compared to a purification by chromatography of affinity, this heat treatment can be a quick, easy, and economically interesting Nit*_*phym*_* purification method for large-scale applications. The molecular mass of Nit*_*phym*_* (37 kDa) is close to molecular masses of other known nitrilases such as Nit09 (36 kDa) (Egelkamp et al., 2020). Its high oligomericity of more than 18 subunits is in accordance with many described nitrilases, the preferred native forms of nitrilases seem to be large aggregates of 6–26 units (Banerjee et al., 2002). Its optimum temperature of approximately 45–55°C is slightly higher than optima reported for many nitrilases from mesophilic organisms (Shen et al., 2021), usually ranging from 30–50°C, such as the one of its close homolog from *Rhodobacter sphaeroides* (Table 2, Uniprot ID: G5DDB2) (71% of protein sequence identity with Nit*_*phym*_*) described to convert also both aromatic and aliphatic nitriles (Wang et al., 2014), or the commerciaized nitrilase from *Arabidopsis thaliana* What is remarkable for a nitrilase from a mesophilic organism is the low effect of temperature on its stability. The stability of Nit*_*phym*_* at 30°C for more than 2 days of incubation is much greater than for other nitrilases with comparable optimal temperature between 40°C and 50°C that generally exhibited a half-life of less than 20 h at 30°C. For example, the well described nitrilase from *Pseudomonas fluorescens* strain EBC191 has similar temperature profile (optima temperature: 45–55°C) but is much more unstable than Nit*_*phym*_* at these temperatures with activity decreasing within 30 min above 40°C (Table 2; Kiziak et al., 2005). More than 50% of activity was lost for the ones from *Ralstonia eutropha* (Fan et al., 2017b) or *Pseudomonas psychrotolerans* (Sun et al., 2016) after storage of less than 10 h at 37–40°C, whereas they displayed broad temperature optima of 30–50°C and 30–60°C, respectively (Table 2). The stability of Nit*_*phym*_* is impressive, with a half-live of 18 h at 60°C, compared to these nitrilases and the one from *Pantoea* sp. (Zhang et al., 2018) or BGC4 from *Paraburkholderia graminis* (Fan et al., 2017a) that show half-lives under 4 h at 50°C. Nit*_*phym*_* is similar to PaCNit reported to maintain more than 50% of its activity after 24 h at 60°C when heterogeneously expressed in *E. coli* (Liu et al., 2019), while it was isolated from *Pannonibacter carbonis*, a proteobacteria with optimum growth at 30–35°C (Xi et al., 2018). Thermoactive nitrilases are commonly obtained from thermophilic and hyperthermophilic microorganisms (Mueller et al., 2006; Chen et al., 2015; Dennett and Blamey, 2016; Cabrera and Blamey, 2017) with activity range usually up to 60–90°C and high stability even at high temperature. For example the nitrilases from the archaea *Pyrococcus* sp M24D13 (Dennett and Blamey, 2016) or *Pyrococcus* sp. MC-FB (Cabrera and Blamey, 2017) displayed half-lives of 8 h at 90°C and 85°C, respectively (Table 2). Nevertheless, significant activity of Nit*_*phym*_* after more than 3 h at 70°C demonstrated a stability that remains much better than those of some other nitrilases from thermophilic organisms (Almatawah et al., 1999). Moreover, high temperatures such as 70–90°C are generally not necessary in industrial processes with nitrilases, where temperatures of 40–60°C are usually sufficient to improve substrate solubility and transfer rates and to reduce the risk of contamination (Elleuche et al., 2014; Choi et al., 2015). However, enzymes from thermophilic organisms are generally not very active at these temperatures (Table 2). Thermostability is also considered as an indicator of enzyme robustness, required feature for protein engineering (Littlechild, 2015; Atalah et al., 2019).

**TABLE 2:** Key characteristics of Nit*_*phym*_* compared to some reported nitrilases overexpressed in *E. coli*.

Name *organism*	Substrate profile and (act. in U⋅mg^–1^ of protein)^*a*^	pH opt. (pH zone with act. >80%)	Temp. opt. in°C (pH zone with act. >80%)	Stability		Notes or example of acid production	References
						
				Temp.	Tolerance to cosolvent^*d*^		
Nit09 *Variovorax boronicumulans*	Phenylacetonitrile (5.2), 2-thiopheneacetonitrile (20.0), aliphatic nitriles: no act.	6.0 (6–8)	50 (45–55)	Loss of 20% act. after 3 weeks at 4°C	5% MeOH, EtOH, DMSO	Inhibition at >10 mM phenylacetonitrile	Egelkamp et al., 2020
blr3397 *Bradyrhizobium japonicum*	Hydrocinnamonitrile (10.0), phenylacetonitrile (2.3), heptanenitrile (2.5)	7.0 (6.5–8.5)	45	Loss of act. after 30 min at 70°C	nr	nr	Zhu et al., 2008
REH16 *Ralstonia eutropha*	3-Cyanopyridine (6.9)^*b*^, cinnamonitrile (55.3)^*b*^, fumaronitrile (248.5)^*b*^	6.5	30 (30–50)	*t*_1/2_ = 10 h at 37°C	25% MeOH, EtOH, iPrOH, DMSO, acetone	100 mM nicotinic acid in 1 h (100 mg⋅mL^–1^ resting cells) in fed batch: 129.2 g⋅L^–1^ in 20.8 h	Fan et al., 2017b
PacNIT *Pannonibacter carbonis*	Wide-spectrum substrate	7.0 (6.5–8.5)	65 (40–70)	*t*_1/2_ > 48 h at 50°C *t*_1/2_ = 24 h at 60–70°C	nr	nr	Liu et al., 2019
NitMC-FB *Pyrococcus* sp.	2-Cyanopyridine (2.3), very low or no act. toward arylaceto- and aliphatic nitriles	7.0 (6.8–7.2)	90 (<10% act. at 50°C)	*t*_1/2_ = 8 h at 90°C	nr	Inhibition at >25 mM 2-cyanopyridine	Mueller et al., 2006
*Rhodobacter sphaeroides* LHS-305	Fumaronitrile, 3-phenylpropinionitrile, 3-cyanopyrifine (*nr*)	7.0 (6.5–8.0)	40 (35–43)	Loss of 38% act. after 11 days at 4°C	10% MeOH, DMSO	Inhibition above 50 mM substrate	Wang et al., 2014
NitA *Pseudomonas fluorescens* EBC191	2-Phenylvaleronitrile (230), phenylacetonitrile (68), 2-thiopheneacetonitrile (205), 3-hexenedinitrile (3.3)^*c*^	5.5–7.5 (5.5–8.5)	50 (45–55)	Loss of act. after 30 min at 40°C loss of 75% act. after 2 weeks at 4°C	nr	Amide formation	Kiziak et al., 2005; Brunner et al., 2018
Nit*_*phym*_ Paraburkholderia phymatum*	*trans*-Cinnamonitrile (9.3), terephthalonitrile (5.0), 2-cyanopyridine (3.0)	6.0–8.5 (5.5–8.5)	50 (40–55)	*t*_1/2_ = 18 h at 60°C	40–50% activity at 15–20% DMSO, MeOH	500 mM 3-phenylpropionic acid in 8 h (0.5 mg⋅mL^–1^ semipurified enzyme)	This work

*^a^Values reported with purified enzyme otherwise stated. ^b^Specific activities reported for 1 mg of dry cell. ^c^Specific activities reported with resting cells. ^d^% of cosolvent is expressed in vol/vol. nr, not reported; act., activity; temp., temperature; opt., optimum.*

Thermoactive nitrilases have generally narrow substrate spectrum (Shen et al., 2021), such as the one from the archaeon *Pyrococcus abyssi* highly specific toward aliphatic dinitriles (Mueller et al., 2006) or the NitMC-FB specific to cyanopyridines (Cabrera and Blamey, 2017; Table 2). On the contrary, Nit*_*phym*_* is active toward various nitriles, especially aromatic/benzylic nitriles, dinitriles, and some aliphatic nitriles. These results are roughly similar to the ones we previously described despite some differences mainly due to solubility issues and inconsistencies occurring in 96-microwell plates screening (Vergne-Vaxelaire et al., 2013). This characterization expands the list of enzymes with broad substrate specificity, which mainly includes PaCNit (Liu et al., 2019; Table 2), NIT278 from *Syntrophobacter fumaroxidans* (Vergne-Vaxelaire et al., 2013), NIT28 from *Sphingomonas witichii* (Vergne-Vaxelaire et al., 2013), NIT191 from *Burkholderia xenovorans* (Vergne-Vaxelaire et al., 2013), BGC4 from *Paraburkholderia graminis* (Fan et al., 2017a), bll6402 and blr3397 from *Bradyrhizobium japonicum* (Zhu et al., 2007, 2008), NitA from *Pseudomonas fluorescens* EBC191 (Brunner et al., 2018; Table 2), and the recently nitrilases mined from *Zobellia galactanivorans*, *Achromobacter insolitus*, and *Cupriavidus necator* (Sunder et al., 2020). The growing number of described nitrilases active on varying classes of nitriles supports the irrelevance of the previously widely used classification of nitrilases based on their main substrates (arylacetonitrilases, aromatic, and aliphatic nitrilases). Interestingly, Nit*_*phym*_* exhibits high activity toward the dinitrile terephthalonitrile rarely tested despite the interest of its corresponding monohydrolyzed product 4-cyanobenzoic acid as precursor of various valuable compounds such as 4-aminomethyl benzoic acid (Zhang et al., 2018). In addition to its preference for *trans*-cinnamonitrile, cyanopyridines, and 3-phenylpropionitrile and its inactivity or very low activity toward arylacetonitriles, Nit*_*phym*_* harbored a substrate spectrum similar to those of PaNIT and REH16. These two enzymes share 71 and 81% protein sequence identity with Nit*_*phym*_*, respectively (Fan et al., 2017b; Zhang et al., 2018). Thus, Nit_*Phym*_ is an attractive alternative to these efficient biocatalysts for the hydrolysis of such nitriles.

In terms of pH and buffer profile, Nit*_*phym*_* has a behavior similar to those of many enzymes with maximum activity around pH 7 (Shen et al., 2021). The activity at this pH is of the same order as those between 6.0 and 8.0, unlike which was observed for the nitrilases PaCNit (Liu et al., 2019) or the one from *Rhodobacter sphaeroides* (Wang et al., 2014) which exhibited narrow pH profiles. For this feature, Nit*_*phym*_* is similar to PaNIT (Zhang et al., 2018) and to the nitrilase from *Pseudomonas fluorescens* strain EBC191 (Kiziak et al., 2005; Table 2). Reasonable activity of 50% maximum activity even around these optimum pHs (pH 5–10), whatever the buffer, ensures more reliable synthesis processes.

Nit*_*phym*_* seemed to distinguish from many reported nitrilases by its high degree of resistance to water miscible organic solvents. Due to the poor solubility of most nitriles, this feature has been frequently studied to get more insights into the capabilities of the biocatalysts to be applied in synthetic reactions, especially at high substrate loadings. Some nitrilases were reported to be stable in alcoholic cosolvent such as MeOH, EtOH, or iPrOH mainly in 1–10% (vol/vol) range, but a residual activity of more than 30% at 15–20% (vol/vol) in MeOH as observed for Nit*_*phym*_* seemed much more unusual (Table 2). For example, the arylacetonitrilase Nit09 displayed less than 10% of residual activity at 20% (vol/vol) with all tested solvent, except glycerol (Egelkamp et al., 2020). The activity reduced only by half in the presence of 20% (vol/vol) DMSO has to be noticed, this solvent being often harmful for enzymes at this ratio (Egelkamp et al., 2020). However, Nit*_*phym*_* was strongly affected by even 5% (vol/vol) of THF, like REH16 from *Ralstonia eutropha*, yet highly tolerant to many solvents even at 25% (vol/vol) (Fan et al., 2017b). The effect of substrate loading was tested on 3-phenylpropionitrile, one of the top substrate of various reported nitrilases mainly showing major activities toward arylacetonitriles, such as Nit09 (0.8 U⋅mg^–1^) (Egelkamp et al., 2020), nitrilase from *P. fluorescens* EBC191 (3.90 U⋅mg^–1^) (Kiziak et al., 2005), BGC4 (Fan et al., 2017a), REH16 (61% of the activity of 3-cyanopyridine) (Fan et al., 2017b) and nitrilases from *Cupriavidus necator*, *Janthinobacterium* sp., and *Acetobacter pasteurianus* (Sunder et al., 2020). This nitrile was chosen for this study, despite it was not the best substrate of Nit*_*phym*_* (Table 1), to attest even more its potential. The complete conversion of the substrate at the high concentration of 500 mM is quite unusual for bacterial nitrilases. Such substrate loadings have been reported with fungal nitrilases (Martínková, 2019) but substrate inhibition was often observed with recombinant archaeon nitrilases at low substrate concentration (∼>12 to 25 mM) (Mueller et al., 2006; Cabrera and Blamey, 2017; Table 2). For bacterial nitrilases the biotransformations/biocatalytic synthesis are with concentrations rather in 100–300-mM range (Fan et al., 2017a). A significant productivity of 8.4 g/(L h) obtained on 30 mL scale at low enzyme loading (0.5 mg⋅mL^–1^ of enzyme purified by heat treatment) with 3-phenylpropionitrile demonstrated its biocatalytic potential. Such high productivity can be expected with its better substrates *trans*-cinnamonitrile and terephthalonitrile. Further amelioration including biotransformation with resting cells and fed batch process, as done with REH16 (1,050 mM of 3-cyanopyridine hydrolyzed in total from 13 batches of 50–100 mM in 20 h) (Fan et al., 2017b), can be considered to reach productivity at least such as the one of 26 g/(L h) obtained using PaNIT with terephthalonitrile (Zhang et al., 2018). It is worthy to note that no amide was detected by UHPLC-UV analyses whatever the conditions tested in this study, demonstrating the absence of nitrile hydratase activity of this enzyme.

In conclusion, using an unusual screening assay based on activity after heat treatment of pre-identified nitrilases, a robust nitrilase Nit*_*phym*_* from mesophilic bacteria was characterized. It exhibits high stability at 30–50°C and broad substrate spectrum with preferred activity toward *trans*-cinnamonitrile, terephthalonitrile, cyanopyridine, and 3-phenylpropionitrile. Together with its tolerance to more than 500 mM of substrate concentration, Nit*_*phym*_* emerges as a promising candidate for industrial hydrolysis of such nitriles, being so a good alternative to PaNIT described as less stable. The outstanding characteristics of Nit*_*phym*_* make it also a potential candidate for efficient degradation of toxic nitriles in effluents. This biocatalyst constitutes also a good template for protein engineering which could be necessary to allow further development (Nigam et al., 2017).

## Data Availability Statement

The original contributions presented in the study are included in the article/Supplementary Material, further inquiries can be directed to the corresponding author/s.

## Author Contributions

VB conceived the project and directed it with CV-V. TB, AM, and CV-V designed the experiments. TB, AM, J-LP, VP, and AD conducted the experiments. TB, AM, J-LP, CV-V, and VB analyzed data. CV-V and VB wrote the manuscript with input from AZ, AM, and J-LP. All authors read and approved the final manuscript.

## Conflict of Interest

The authors declare that the research was conducted in the absence of any commercial or financial relationships that could be construed as a potential conflict of interest.
