# Supplementary material for: Purification and Characterization of Nitphym, a Robust Thermostable Nitrilase From Paraburkholderia phymatum
Source: Front Bioeng Biotechnol. 2021 Jul 1;9:686362. doi: 10.3389/fbioe.2021.686362 (PMC8280356; doi:10.3389/fbioe.2021.686362)
Supplement: Supplementary file 1 [file Data_Sheet_1.PDF]

Supplementary Material

Purification and Characterization of Nit$_{phym}$, a Robust Thermostable Nitrilase from *Paraburkholderia phymatum*

Thomas Bessonnet [1]†, Aline Mariage[1]†, Jean-Louis Petit[1], Virginie Pellouin[1], Adrien Debard[1], Anne Zaparucha[1], Carine Vergne-Vaxelaire[1]* and Véronique de Berardinis[1]*

† These authors have contributed equally to this work.

[1]Génomique Métabolique, Genoscope, Institut François Jacob, CEA, CNRS, Univ Evry, Université Paris-Saclay, 91057 Evry, France

*** Correspondence:**
Carine Vergne-Vaxelaire, Véronique de Berardinis
carine.vergne@genoscope.cns.fr, vberard@genoscope.cns.fr

1 Supplementary Figures

A

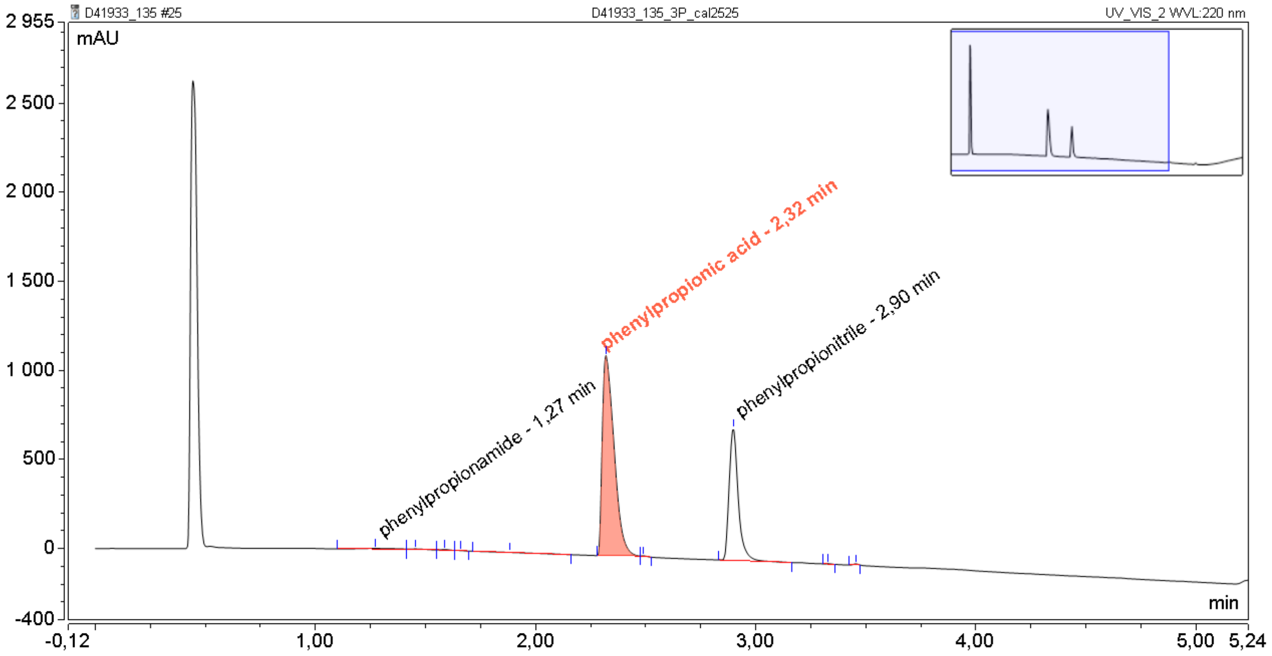

B

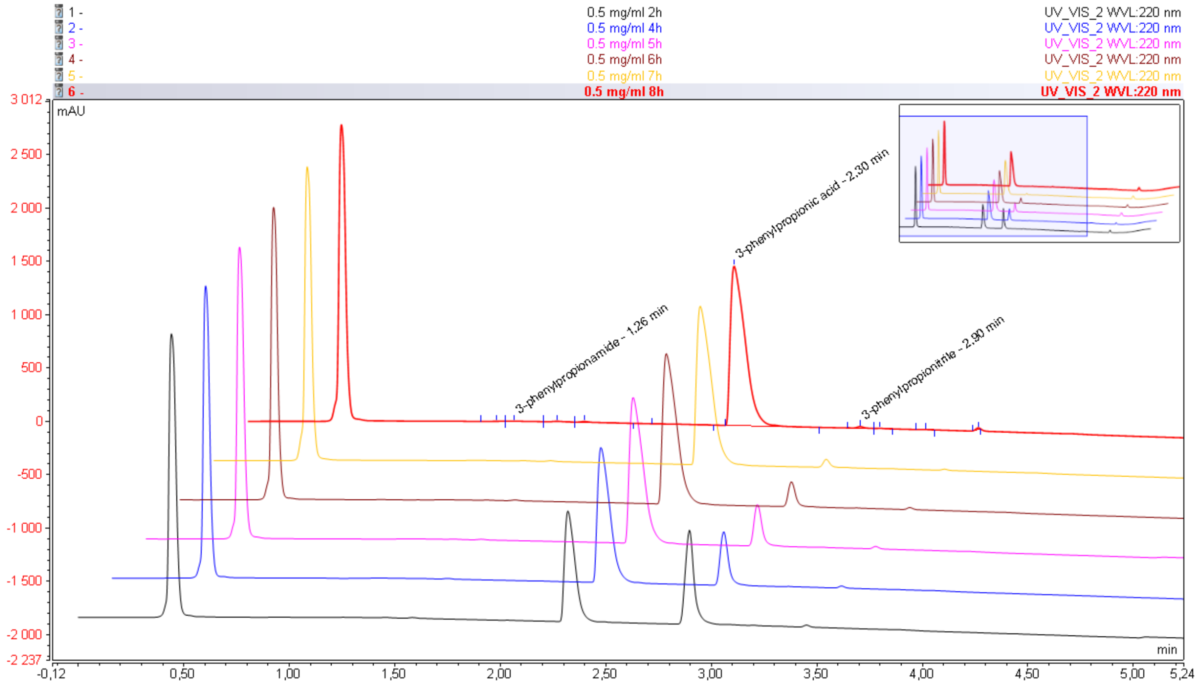

C

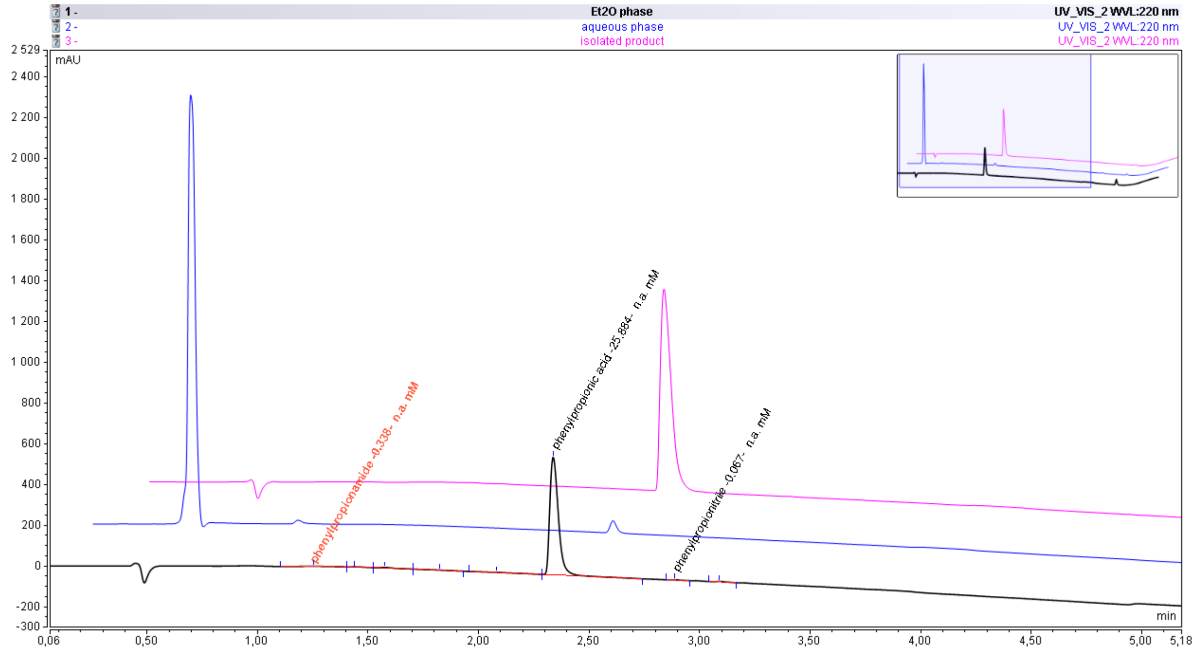

Supplementary Figure 1. UHPLC-UV chromatograms of standard 3-phenylpropionic acid and 3-phenylpropionitrile (**A**), preparative scale reaction of 500 mM of 3-phenylpropionitrile with Nit*phym* purified by heat treatment at 2 h – 8 h (**B**), Et_2O organic phase during extraction procedure, aqueous phase during extraction procedure and isolated product (**C**).

A

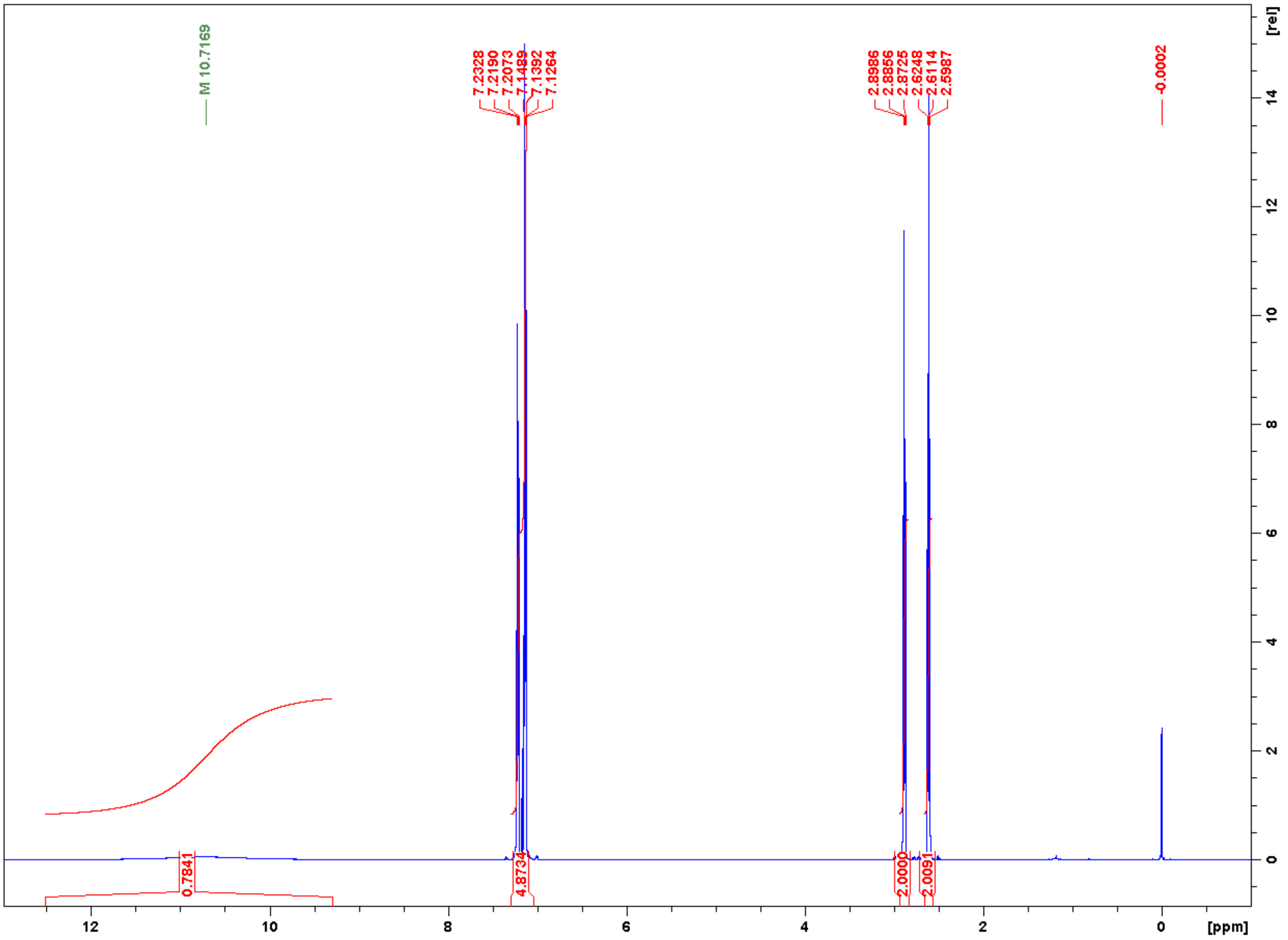
M 10.7169
7.2328
7.2190
7.2073
7.1483
7.1392
7.1264
2.8986
2.8856
2.8725
2.6248
2.6114
2.5987
-0.0002
0.7841
4.8734
2.0000
2.0091
[rel]
[ppm]

B

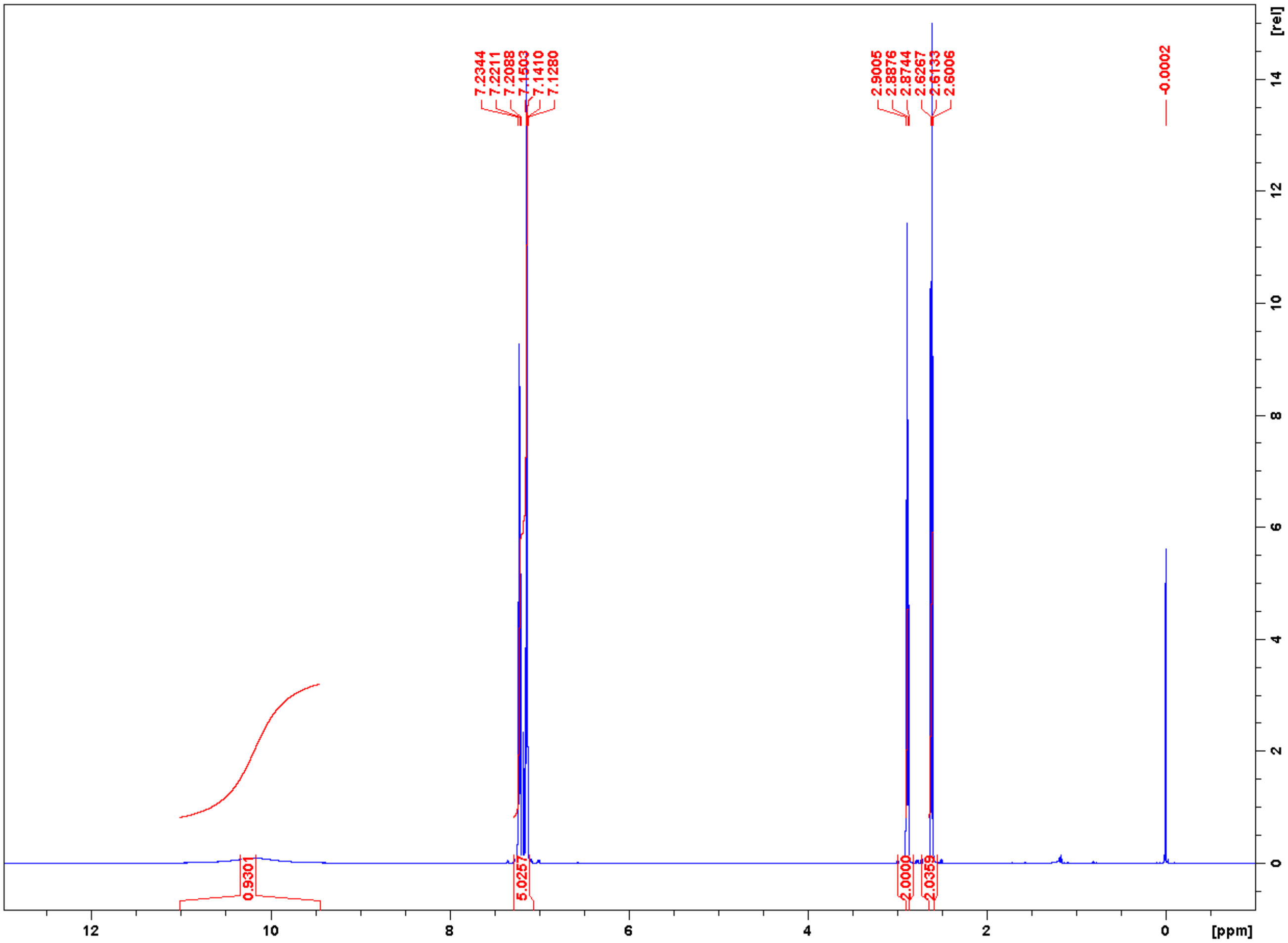

Supplementary Figure 2. ^{1}H NMR spectra of commercial (**A**) and synthesized (**B**) 3-phenylpropionic acid.

A

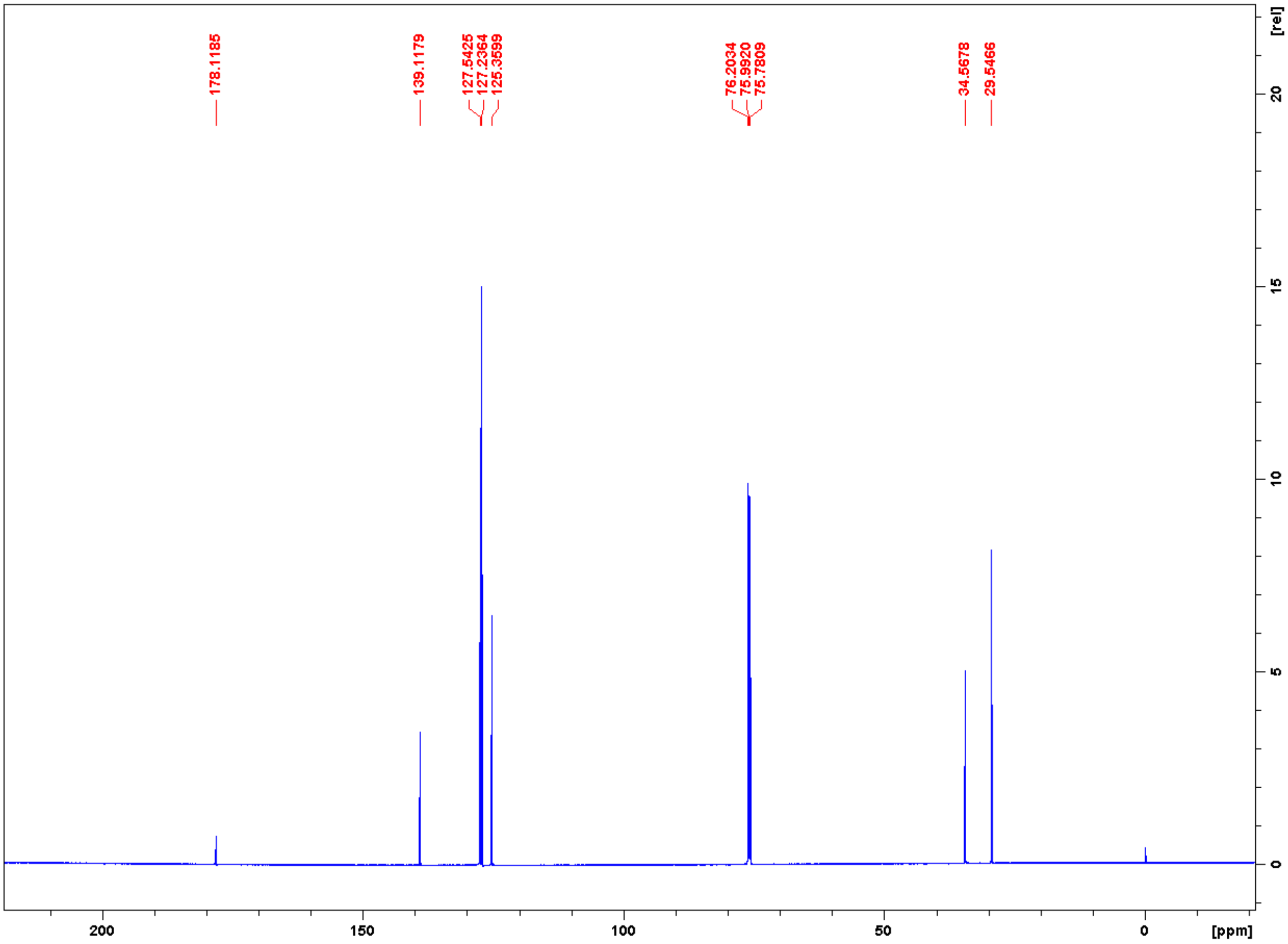
178.1185
139.1179
127.5425
127.2364
125.3599
76.2034
75.9920
75.7809
34.5678
29.5466
[rel]
20
15
10
5
0
200
150
100
50
0
[ppm]

B

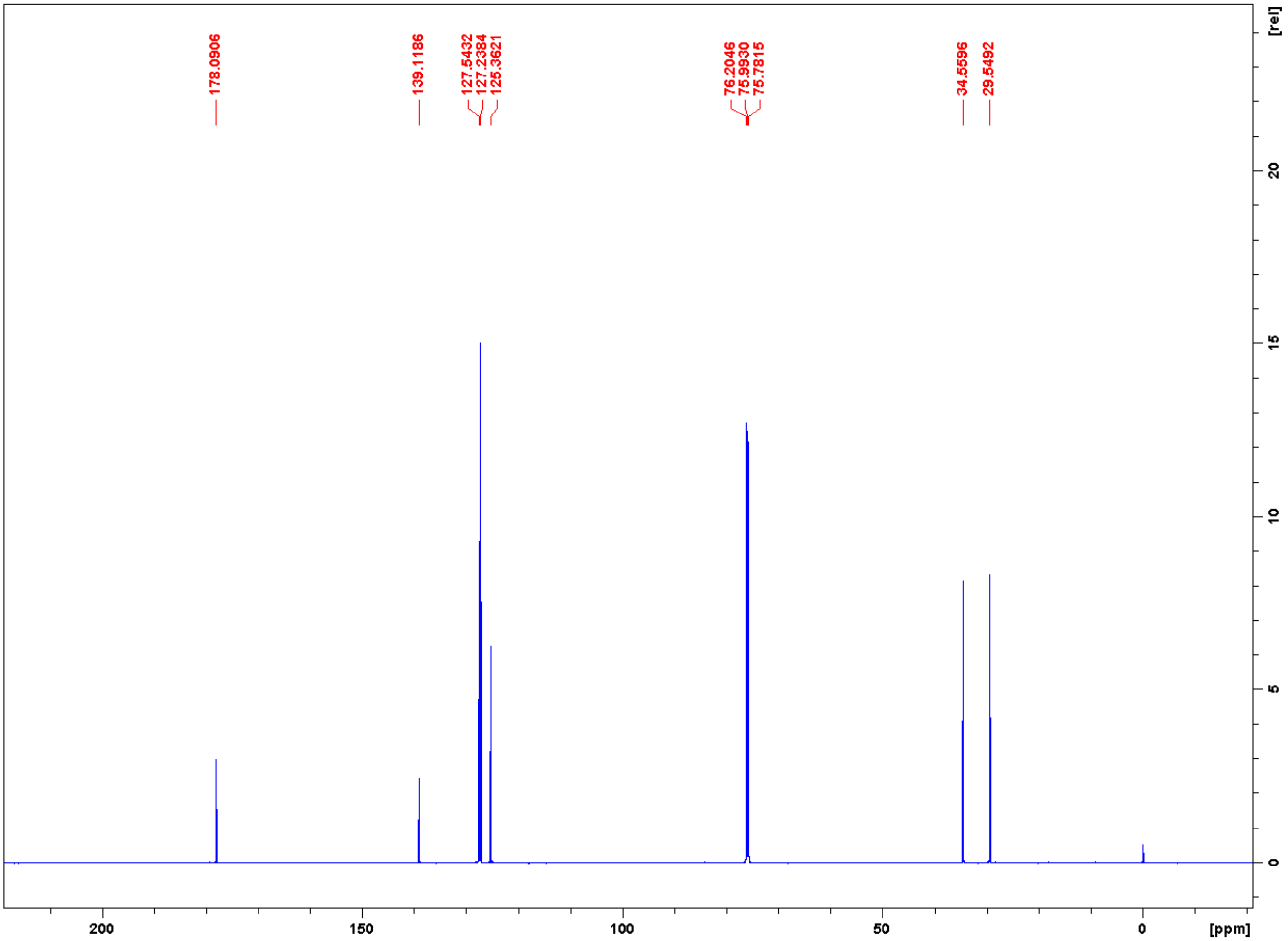

Supplementary Figure 3. ^{13}C NMR spectra of commercial (**A**) and synthesized (**B**) 3-phenylpropionic acid.
